# An Early Single-Center Case Series Experience With a Recently Introduced Formulation of Inhaled Treprostinil (Yutrepia)

**DOI:** 10.7759/cureus.114762

**Published:** 2026-08-18

**Authors:** Christopher R Moore, Munish Sharma, Andrew I Kim, Shekhar Ghamande, Peter Yau

**Affiliations:** 1 Pulmonary and Critical Care, Baylor Scott and White Medical Center - Temple, Temple, USA

**Keywords:** group 1 ph, group 3 ph, inhaled treprostinil, pulmonary hypertension, pulmonary vascular disease, yutrepia

## Abstract

Background: Treprostinil inhalation powder produced using PRINT technology (LIQ861; delivered via the RS00 Model 8 dry powder inhaler; Yutrepia) is an FDA-approved inhaled treprostinil therapy for pulmonary arterial hypertension (PAH) and pulmonary hypertension associated with interstitial lung disease (PH-ILD). Evidence from the INSPIRE phase 3 open-label study supported long-term safety and tolerability; however, real-world implementation data remain limited.

Methods: We conducted a retrospective, single-center observational case series of consecutive patients initiated on Yutrepia in routine clinical practice between June and December 2025. Our cohort included group 1, group 3, and mixed group 1 and 3 treated at our Pulmonary Hypertension Center of Excellence. The primary objective of this study was to evaluate the real-world safety and tolerability of Yutrepia, with particular attention to airway-related adverse events (AEs), dose modification, and treatment discontinuation in routine clinical practice. Secondary outcomes included initial and maintenance doses achieved. Additional AEs recorded include dizziness, headache, nausea, hypotension, hypertension, tachycardia, fatigue, and hypoxemia. All data were calculated using a Wilson 95% confidence interval (CI) given the small sample size.

Results: Twenty-two patients were included (mean age: 66.4 ± 13.8 years; 16 (72.7%) female patients). PH etiology was group 1 in 13 of 22 patients (59.1%), group 3 in 3 of 22 (13.6%), and mixed group 1 and 3 in 6 of 22 (27.3%). Of the 22 patients, 17 (77.27%) were on background therapy: 11 of 13 patients (84.62%) in group 1, 1 of 3 patients (33.33%) in group 3, and 5 of 6 patients (83.33%) in mixed group 1 and 3. Airway-specific AEs were documented in 8 of 22 patients (36.4%; 95% CI: 19.7%-57.0%). Shortness of breath occurred in 5 of 22 (22.7%; 95% CI: 10.1%-43.4%), cough occurred in 3 of 22 (13.6%; 95% CI: 4.7%-33.3%), and no cases of throat irritation were documented (0.0%; 95% CI: 0.0%-14.9%). Dose modification due to any AE occurred in 9 of 22 patients (40.9%; 95% CI: 23.3%-61.3%), and no patients required dose modification or discontinuation because of airway-related AEs (0.0%; 95% CI: 0.0%-14.9%). Due to the retrospective design, causal attribution of individual events to Yutrepia could not always be established.

Conclusions: In this retrospective, single-center case series of Yutrepia, no airway-related dose modifications or discontinuations were documented; however, the 95% confidence interval (0.0%-14.9%) indicates that clinically relevant event rates cannot be excluded given the small sample size. Overall, however, 54.5% of patients experienced an adverse event, 40.9% required dose modification, and 36.4% discontinued treatment because of an adverse event. These findings should not be interpreted as indicating that Yutrepia was broadly well tolerated. The observed frequencies of airway-related adverse events were descriptively lower for some events than those reported in selected previous studies of inhaled treprostinil, but direct comparisons cannot be made because of differences in study populations, designs, follow-up, and adverse event ascertainment. Given the small sample size and retrospective design, these findings are hypothesis-generating, and larger prospective studies are needed to define persistence, tolerability, and longer-term clinical outcomes.

## Introduction

Pulmonary hypertension (PH) is a chronic and often progressive disease involving abnormal elevation of pressure within the pulmonary circulation. It is currently defined hemodynamically by a mean pulmonary artery pressure (mPAP) of at least 20 mmHg at rest on right heart catheterization. PH is not a singular disease process, but rather a broad clinical syndrome composed of multiple conditions with distinct underlying mechanisms and treatment considerations. For this reason, PH is divided into five clinical groups based on shared pathophysiology and hemodynamic profiles. Over time, persistent elevation in pulmonary vascular pressures can result in worsening right ventricular strain, functional decline, reduced quality of life, and eventual right heart failure. Although advances in targeted therapies have improved outcomes in select patient populations, pulmonary hypertension continues to carry significant morbidity and mortality, and optimization of treatment tolerability and long-term management remains an important area of ongoing study. The clinical course is particularly challenging in patients with pulmonary arterial hypertension (PAH) and selected forms of PH associated with parenchymal lung disease, where therapeutic decisions must balance efficacy, tolerability, disease severity, comorbidity burden, and patient ability to adhere to complex treatment regimens [1-5].

The prostacyclin pathway remains a central therapeutic target in PH [5]. Prostacyclin analogues and prostacyclin receptor agonists can improve pulmonary vascular tone, inhibit vascular smooth muscle proliferation, and support right ventricular-pulmonary vascular coupling through reductions in pulmonary vascular resistance [6]. In clinical practice, traditional prostacyclin delivery systems impose meaningful burdens. Nebulized inhaled treprostinil provides a nonparenteral option, but airway-related adverse effects, including cough and throat irritation, in a population with these at baseline can limit tolerability for some and have a clearly documented history of affecting compliance. In one large observational safety study, cough occurred in 18% and throat irritation in 6% of patients receiving inhaled treprostinil [7].

Dry powder inhaled treprostinil was developed to simplify inhaled prostacyclin delivery while preserving targeted pulmonary administration based upon the physiologic concept of V/Q mismatch [8,9]. Treprostinil inhalation powder (Yutrepia) was evaluated in the INSPIRE open-label study, which supported long-term safety and tolerability in PAH, and the formulation subsequently received FDA approval for PAH and PH-ILD. A portable dry powder platform may reduce treatment complexity compared with nebulized therapy and may be attractive for patients transitioning from older inhaled systems or for those in whom parenteral therapy is not feasible or acceptable [7].

Randomized and prospective trial data provided critical evidence but do not fully capture the heterogeneity encountered in routine PH practice. Patients treated at specialty centers often have overlapping PH phenotypes, variable functional status, multiple comorbidities, prior exposure to prostacyclin therapy, differing goals of care, and differing reactions to medications. Real-world data specifically characterizing post-marketing safety, tolerability, and treatment persistence with Yutrepia remain limited, particularly regarding airway-related adverse events (AEs) across the heterogeneous PH populations encountered outside clinical trials.

We therefore performed a single-center retrospective case series evaluating early clinical experience with a newer form of inhaled treprostinil powder produced using PRINT technology (LIQ861; delivered via the RS00 Model 8 dry powder inhaler; Yutrepia). The objectives were to describe airway-related adverse events and subsequent treatment discontinuation, dose modification, and available short-term outcomes after initiation.

## Materials and methods

In our retrospective, single-center observational case series conducted between June and December of 2025, patients were eligible for inclusion if they had a clinical diagnosis of group 1 pulmonary arterial hypertension (PAH), group 3 pulmonary hypertension (PH), or mixed group 1 and group 3 PH; were evaluated or managed at the study center; and had documented initiation of inhaled treprostinil dry powder in the medical record. Patients were excluded if the medication was prescribed but never initiated; they had a PH classification other than group 1, group 3, or mixed group 1 and group 3 PH; patients with group 2 PH were not included in the study cohort; follow-up documentation after treatment initiation was unavailable after initiation; or key baseline data were insufficient to confirm eligibility.

Clinical data were abstracted from the electronic medical record using a standardized data collection template by two reviewers (CM and MS), with extracted data verified by a third reviewer (AK). Extracted variables included demographics, pulmonary hypertension classification, baseline functional class, 6-minute walk distance, natriuretic peptide values, echocardiographic and hemodynamic parameters when available, background pulmonary hypertension therapies, prior inhaled prostacyclin exposure, indication for therapy initiation, starting dose, titration pattern, maintenance dose, adverse events, dose modifications, and treatment discontinuation. Follow-up data were collected from clinic notes, testing reports, medication documentation, and telephone encounters when available. Follow-up was available through the study period (June-December 2025); depending on the date of Yutrepia initiation, observed follow-up ranged from approximately 1-7 months. Median follow-up duration was not systematically calculated.

The primary objective was to describe real-world tolerability of Yutrepia, with specific evaluation of airway- and non-airway-related adverse events including neurologic, cardiovascular, general, and airway symptoms and their influence on medication titration or discontinuation. Demographic data, including patient characteristics, treatment patterns, and treatment dosing, were collected. Safety endpoints included any documented adverse event, airway-related adverse events, dose reduction or interruption due to adverse events, and discontinuation due to adverse effects as determined by investigator clinical judgment rather than a predefined causality algorithm, recognizing that dyspnea in this population may reflect underlying disease progression rather than drug-related toxicity. Adverse events were ascertained through retrospective review of clinical documentation (progress notes, telephone encounters, and medication records) rather than a prospective, protocolized assessment.

Data management and statistical analyses were performed using Microsoft Excel for Microsoft 365 version 2606 (Microsoft Corporation, Redmond, WA). Analyses were limited to descriptive statistics; given this and the small sample size, formal comparative hypothesis testing was not performed. Categorical variables were summarized as counts and percentages using available case denominators. For binomial proportions, 95% confidence intervals (CIs) were calculated using the Wilson score method to provide stable interval estimates in the setting of a small sample size [10]. The Microsoft Excel spreadsheet syntax used to calculate Wilson score confidence intervals was generated with the assistance of ChatGPT (OpenAI, San Francisco, CA). The mathematical logic and algebraic accuracy of the AI-generated formulas were manually reviewed and verified by the authors before implementation to ensure the accuracy and integrity of the statistical calculations.

This study was conducted under institutional review board oversight according to local policies for retrospective chart review. The requirement for informed consent was waived or not required as determined by the institutional review board because the study involved retrospective review of existing clinical data and posed minimal risk to participants. Data were handled in accordance with institutional privacy and confidentiality requirements. Data were extracted using a standardized data collection template developed by the study authors specifically for this retrospective case series (Appendices). All patient data were deidentified following extraction and before analysis, and no directly identifying patient information was retained in the analytic dataset.

## Results

**Figure 1:**
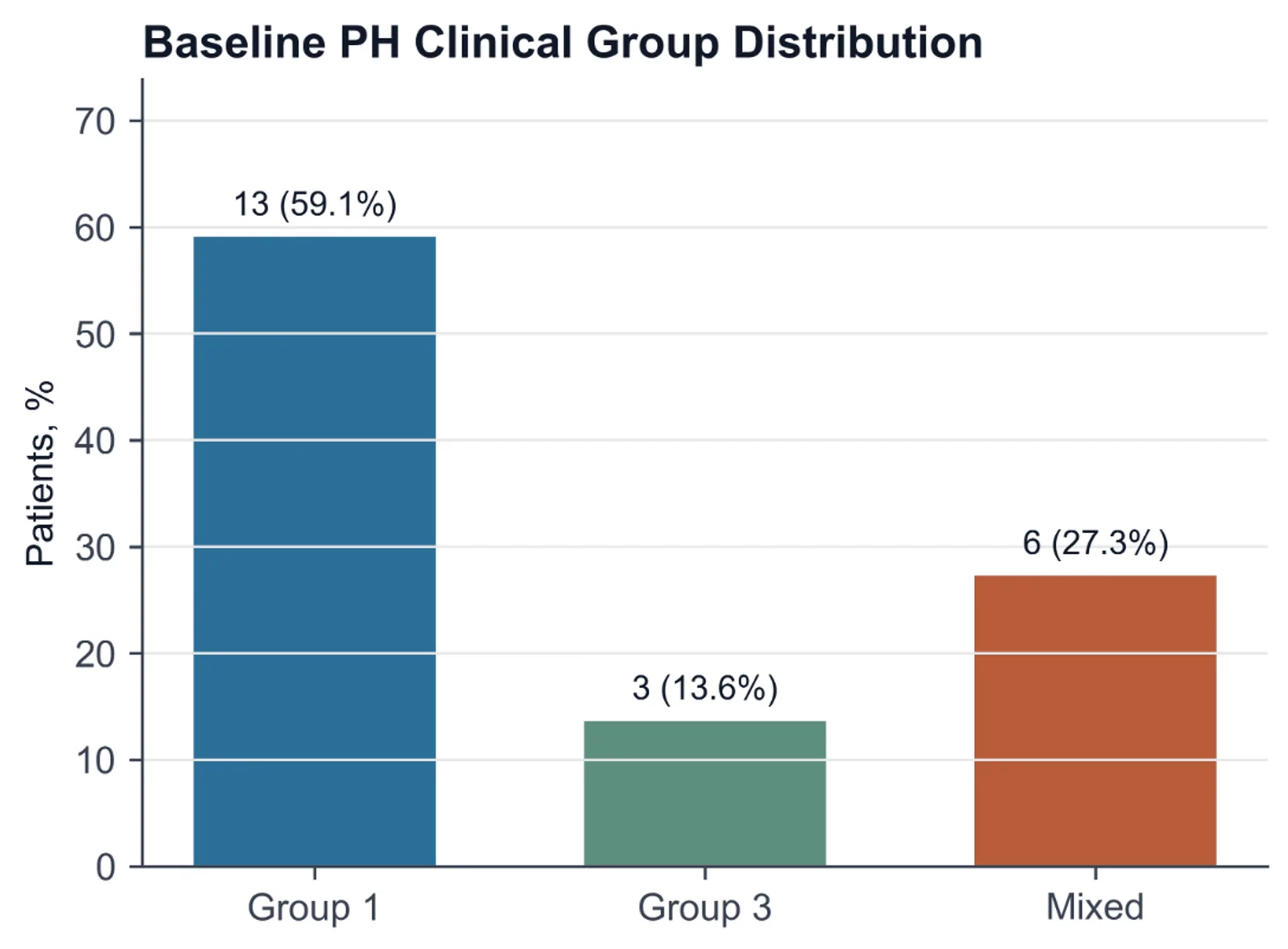
Distribution of baseline pulmonary hypertension clinical classifications within the study cohort Among the 22 patients included in the study, 13 (59.1%) had group 1 PH, 3 (13.6%) had group 3 PH, and 6 (27.3%) had mixed group 1 and group 3 PH. Bars represent the percentage of the total study cohort, with the corresponding number and percentage of patients displayed above each bar. PH: pulmonary hypertension, mixed: group 1 pulmonary hypertension and group 3 pulmonary hypertension

Twenty-two patients met criteria for inclusion. Baseline characteristics are presented in Table 1. The cohort was older, predominantly female, and clinically heterogeneous, with group 1 PH in 13 patients (59.1%), group 3 PH in 3 patients (13.6%), and mixed group 1 and 3 physiology in 6 patients (27.3%) (Figure 1). Among patients with documented WHO functional class, most had at least moderately symptomatic disease; 9 of 17 patients were class III or IV. Baseline exercise capacity was limited, with a mean 6-minute walk distance of 214.3 ± 111.4 m among those with available testing.

**Table 1:** Baseline demographic and pulmonary hypertension characteristics of the study cohort Characteristics included age, sex, and PH classification (group 1, group 3, or mixed group 1 and group 3). Age is presented as mean ± SD in years. Sex and PH classification are presented as number (%). The total study cohort included 22 patients (N = 22). N: total study population, no.: number of patients within the specified category, PH: pulmonary hypertension, SD: standard deviation

Characteristics	Overall cohort (N = 22)
Age, years (mean ± SD)	66.4 ± 13.8
Female (no. (%))	16 (72.7)
Group 1 PH (no. (%))	13 (59.1)
Group 3 PH (no. (%))	3 (13.6)
Mixed group 1 and 3 (no. (%))	6 (27.3)

Dosing and treatment outcomes are summarized in Table 2. The median initial dose was 26.5 mcg QID, and the mean initial dose was 56.6 mcg QID. The discrepancy between median and mean starting dose can be attributed to patients transitioning from a different formulation of inhaled treprostinil who were initiated on a higher, equivalent dose of Yutrepia. Equivalent starting doses for these patients were determined using a published dose-comparison reference between Yutrepia and another FDA-approved inhaled treprostinil formulation, which provides breath-for-breath and cartridge-for-capsule dose equivalency based on total powder load. The conversions were not based on a linear extrapolation performed by the study authors. The median maintenance dose was 106 mcg QID, with a mean maintenance dose of 101.8 mcg QID. Dose modification due to any adverse event occurred in 9 of 22 patients (40.9%; 95% CI: 23.3%-61.3%), whereas airway-related dose modification was 0 of 22 patients (0.0%; 95% CI: 0.0%-14.9%). Further categorization of the 9 dose modifications by specific non-airway adverse event was not performed in this dataset. Discontinuation for any reason occurred in 9 of 22 patients (40.9%; 95% CI: 23.3%-61.3%); 8 patients discontinued because of an adverse event, 1 patient discontinued because of inefficacy, and 0 patients discontinued because of airway-related adverse events (0.0%; 95% CI: 0.0%-14.9%). We did not further categorize the 8 AE-related discontinuations in this dataset.

**Table 2:** Initial and maintenance dosing characteristics, dose modifications, and treatment discontinuations during Yutrepia therapy Data are presented as mean, median, or number (%), as applicable. Percentages were calculated using the total study population (N = 22). Airway-related AEs included cough, dyspnea, and throat irritation. The 95% confidence intervals for categorical outcomes were calculated using the Wilson score method. Attribution of dyspnea specifically to airway intolerance should be interpreted cautiously, as dyspnea in patients with advanced PAH/PH-ILD may reflect underlying disease rather than treatment-related airway irritation. AE: adverse event, CI: confidence interval, mcg: micrograms, N: total study population, no.: number of patients with the specified outcome, QID: four times daily, PAH: pulmonary arterial hypertension, PH-ILD: pulmonary hypertension associated with interstitial lung disease

	Result (N = 22)	95% CI
Dosing (mcg QID)		
Initial median dose	26.5	-
Initial mean dose	56.6	-
Maintenance median dose	106	-
Maintenance mean dose	101.8	-
Dose modification (no. (%))		
Any AE	9 (40.9)	23.3-61.3
Dose modification (airway AE)	0 (0.0)	0.0-14.9
Discontinuation (no. (%))		
Discontinuation overall	9 (40.9)	23.3-61.3
Due to any AE	8 (36.4)	19.7-57.0
Due to airway AE	0 (0.0)	0.0-14.9
Due to inefficacy	1 (4.5)	0.8-21.8

**Figure 2:**
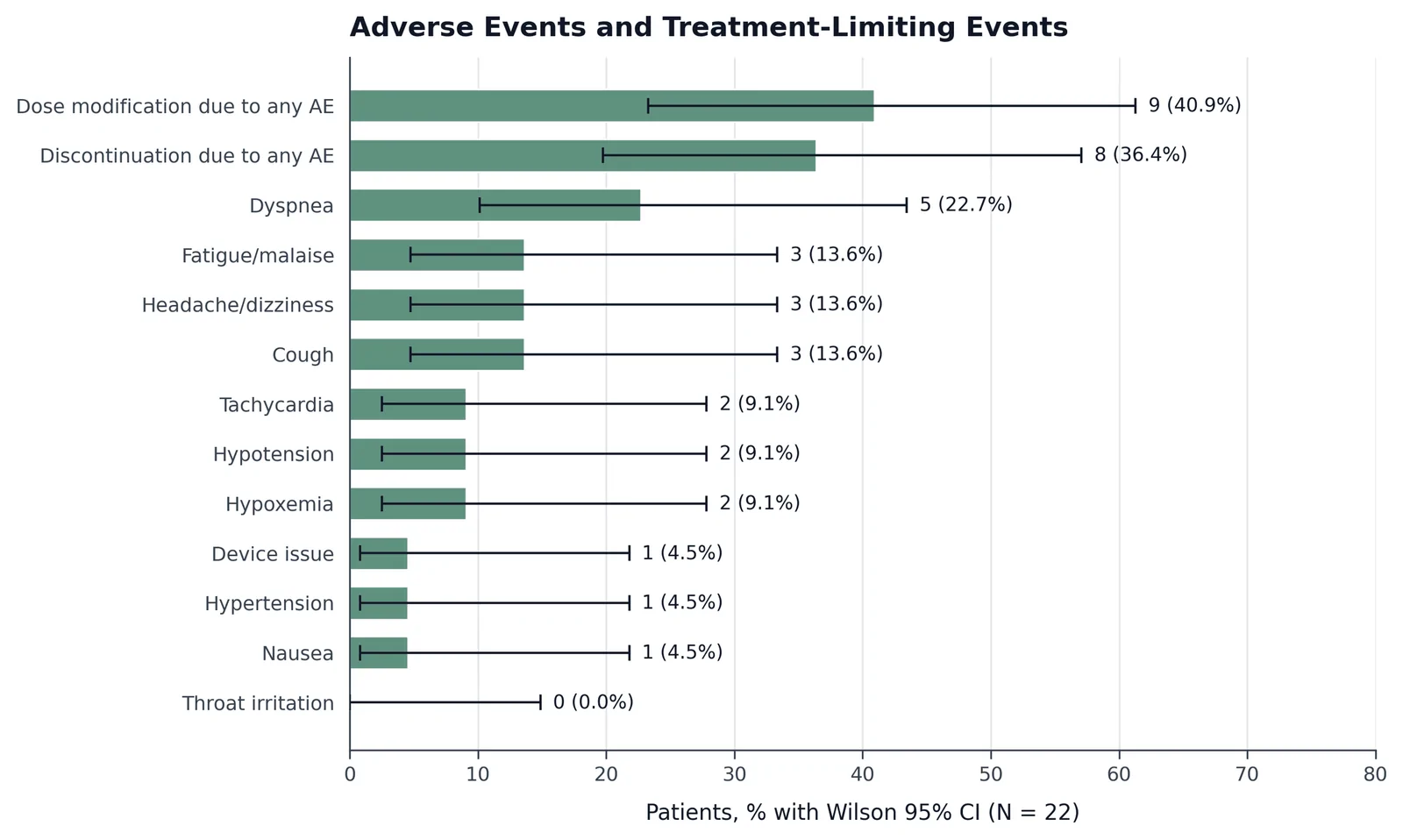
Frequency of adverse events and treatment-limiting events following initiation of Yutrepia therapy Bars represent the percentage of patients experiencing each event or treatment outcome, with the corresponding number and percentage of patients displayed adjacent to each bar. Error bars represent Wilson 95% confidence intervals. Dose modification due to any adverse event occurred in 9 patients (40.9%), and treatment discontinuation due to any adverse event occurred in 8 patients (36.4%). Individual adverse events included dyspnea, fatigue/malaise, headache/dizziness, cough, tachycardia, hypotension, hypoxemia, device-related issues, hypertension, nausea, and throat irritation. Patients may have experienced more than one adverse event; therefore, event categories were not mutually exclusive, and percentages may not sum to 100%. Percentages were calculated using the total study cohort (N = 22). AE: adverse event, CI: confidence interval, N: total study population

Adverse events are summarized in Table 3 and Figure 2. Any adverse event was documented in 12 of 22 patients (54.5%; 95% CI: 34.7%-73.1%), most commonly respiratory or airway symptoms, neurologic symptoms, fatigue, and malaise, with cardiovascular events reported less frequently. Neurologic adverse events were recorded separately as dizziness that occurred in 3 patients (13.6%; 95% CI: 4.7%-33.3%) and headache also occurring in 3 patients (13.6%; 95% CI: 4.7%-33.3%). These were not combined into a single category, and overlap between the two symptoms within individual patients was not separately characterized. In a prior observational study evaluating inhaled treprostinil, 395 of 1,333 patients (30%) prematurely discontinued the study, including 205 of 666 patients (31%) receiving inhaled treprostinil and 190 of 667 patients (28%) in the control group [11]. Reported reasons for premature discontinuation in the inhaled treprostinil and control groups, respectively, included death (14% versus 11%), loss to follow-up (3% versus 4%), withdrawal of consent (2% versus 2%), respiratory-related adverse events (<1% versus <1%), and other reasons (11% versus 12%) [11]. Among patients receiving inhaled treprostinil, cough occurred in 122 of 666 patients (18%), dyspnea in 55 of 666 patients (8%), and throat irritation in 39 of 666 patients (6%) [11]. In a single-center retrospective cohort study evaluating previous formulations of inhaled treprostinil, 67 of 270 patients (24.8%) were no longer receiving therapy at 3 months, including 44 patients (16.3% of the overall cohort) who discontinued treatment [12]. Among the 44 patients who discontinued therapy, dyspnea or hypoxemia was the most frequently reported reason for discontinuation, occurring in 19 patients (43.2%), while cough was reported as the reason for discontinuation in 3 patients (6.8%) [12]. At 6 months, 105 of 270 patients (38.9%) were no longer receiving therapy, including 58 patients (21.5% of the overall cohort) who discontinued treatment [12]. Among the 58 patients who discontinued therapy by 6 months, dyspnea or hypoxemia was reported in 19 patients (32.8%), while cough was reported in 4 patients (6.9%) [12]. These comparative figures are presented descriptively only. Differences in study design, population characteristics, follow-up duration, adverse event ascertainment methodology, and treprostinil formulation preclude any inference of a comparative safety advantage for Yutrepia.

**Table 3:** Adverse events reported following initiation of Yutrepia therapy Adverse events were categorized as respiratory or airway, neurologic, cardiovascular, or general/other events. Respiratory or airway events included shortness of breath, cough, and throat irritation; neurologic events included dizziness and headache; cardiovascular events included hypotension, hypertension, and tachycardia; and general/other events included hypoxemia, fatigue/malaise, and nausea. Data are presented as number (%), with percentages calculated using the total study cohort (N = 22). Patients may have experienced more than one adverse event; therefore, individual adverse event categories are not mutually exclusive, and percentages may not sum to 100%. The 95% confidence intervals were calculated using the Wilson score method. AE: adverse event, CI: confidence interval, N: total study population, no.: number of patients with the specified adverse event

Adverse event	Events reported (N = 22)	95% CI
Any AE (no. (%))	12 (54.5)	34.7-73.1
Respiratory/airway (no. (%))		
Dyspnea	5 (22.7)	10.1-43.4
Cough	3 (13.6)	4.7-33.3
Throat irritation	0 (0.0)	0.0-14.9
Neurologic (no. (%))		
Dizziness/headache	3 (13.6)	4.7-33.3
Cardiovascular (no. (%))		
Hypotension	2 (9.1)	2.5-27.8
Hypertension	1 (4.5)	0.8-21.8
Tachycardia	2 (9.1)	2.5-27.8
General/other (no. (%))		
Hypoxemia	2 (9.1)	2.5-27.8
Fatigue/malaise	3 (13.6)	4.7-33.3
Nausea	1 (4.5)	0.8-21.8

## Discussion

In this single-center retrospective case series, Yutrepia was used in a clinically complex PH population with substantial baseline symptom burden and frequent background PH therapy. The high overall rates of adverse events, dose modification, and discontinuation observed in this cohort temper interpretation of tolerability beyond the airway-specific findings. The cohort included patients with group 1 PH, group 3 PH, and mixed physiology, reflecting the heterogeneity encountered in contemporary PH practice (Table 1, Figure 1). Most patients had impaired functional status or limited exercise capacity at baseline. These features are important when interpreting treatment persistence and tolerability, because many patients considered for inhaled prostacyclin therapy have advanced cardiopulmonary disease, competing comorbidities, and limited physiologic reserve [13].

The observed dosing patterns and adverse event profile seen in our study provide preliminary descriptive information about the practical implementation of Yutrepia in routine clinical practice. Starting and maintenance doses varied across patients, including individuals transitioned from other inhaled treprostinil formulations; therefore, dosing patterns should be interpreted descriptively rather than as a standardized titration strategy (Table 2). This pattern is clinically relevant because real-world titration may differ from structured trial protocols and reflects how clinicians balance symptom burden, adverse effects, and treatment goals in routine practice. These data complement the prospective safety and tolerability experience reported in INSPIRE while providing a pragmatic center-level view of early clinical use. We recommend that these comparisons should be interpreted cautiously because the differences in study design, patient characteristics, and follow-up duration preclude direct comparison across studies.

Tolerability was a central finding. More than one-half of patients had a documented adverse event, and adverse events commonly led to dose modification or treatment discontinuation except for specific airway-related adverse events (Table 3, Figure 2). Respiratory symptoms were clinically important and our central focus, but structured airway-specific dose modification or airway-specific discontinuation was not observed in any patients. These observations should be interpreted cautiously because retrospective adverse event ascertainment is dependent on clinical documentation and may not distinguish drug intolerance from symptoms related to underlying cardiopulmonary disease.

Clinical outcome assessment was limited by incomplete structured follow-up testing. There were no emergency department visits or hospitalizations after treatment initiation in this cohort during the available follow-up period. Similarly, missing 3-month 6-minute walk distance and natriuretic peptide data limited assessment of functional or biomarker response. These limitations are typical of early retrospective case series but are clinically relevant: they highlight the need for prospective workflows that capture standardized follow-up measures when new PH therapies are implemented in practice.

This study has several limitations. The sample size was small, the analysis was descriptive, and all patients were managed at a single center, limiting generalizability. Treatment selection, dosing, titration, and follow-up were not standardized. Missing data were not imputed. Medication class assignment depended partly on abstraction from clinical medication text, and adverse events were captured from routine documentation rather than protocolized assessment. The study was not designed to estimate treatment efficacy or compare Yutrepia with other prostacyclin delivery systems such as subcutaneous and intravenous.

Despite these limitations, the case series provides practical early experience with Yutrepia therapy in a real-world PH population. These findings provide preliminary information regarding early implementation and treatment persistence with Yutrepia. Larger studies are required to identify patient characteristics associated with tolerability and persistence, and close early follow-up, proactive adverse event management, and careful documentation of objective response measures remain important considerations for clinical implementation.

## Conclusions

This study included heterogeneous symptomatic patients with PH on background therapy. Yutrepia required no airway-related dose modifications or discontinuations. However, the small sample size and wide confidence interval (0.0%-14.9%) prevent us from ruling out significant airway-related events. Due to the high overall incidence of adverse events, these findings do not demonstrate broad tolerability. Design and population disparities limit direct comparisons with prior inhaled treprostinil trials. These hypothesis-generating results require confirmation in larger, prospective, multicenter studies.

## Appendices

Table 4 shows the data collection template used to extract data for the present study.

**Table 4:** Standardized data collection template used for retrospective chart review This author-developed template was used to systematically collect deidentified patient-level data from the electronic health record. Extracted variables included baseline demographic characteristics; pulmonary hypertension clinical classification; baseline functional, laboratory, echocardiographic, and hemodynamic characteristics; background pulmonary hypertension therapies; prior inhaled treprostinil exposure; indication for Yutrepia initiation; initial and maintenance dosing; dose titration; device-related issues; adverse events; dose modifications; treatment discontinuation and reasons for discontinuation; treatment duration; healthcare utilization; and available follow-up clinical data. Binary variables were coded as 0 or 1, as specified within the template. The template was developed by the study authors specifically for this retrospective case series. No directly identifying patient information was included in the data collection instrument. 6MWD: 6-minute walk distance, AE: adverse event, BL: baseline, BMI: body mass index, BNP: B-type natriuretic peptide, CI: cardiac index, CO: cardiac output, DC: discontinuation, EF: ejection fraction, ER: emergency room, FU: follow-up, NT-proBNP: N-terminal pro-B-type natriuretic peptide, O₂: supplemental oxygen, PA: pulmonary artery, PAH: pulmonary arterial hypertension, PCWP: pulmonary capillary wedge pressure, PH: pulmonary hypertension, PVR: pulmonary vascular resistance, QID: four times daily, RA: right atrial, RHC: right heart catheterization, RV: right ventricular, RVSP: right ventricular systolic pressure, TAPSE: tricuspid annular plane systolic excursion, WHO-FC: World Health Organization functional class

Study ID	Age	Sex (0 = female, 1 = male)	Ethnicity (0 = White, 1 = Black, 2 = Hispanic, 3 = Asian	Height (cm)	Weight (kg)	BMI	PAH group (etiology) group 1 (1 = yes, 0 = no)	PAH group 3	PAH group mixed	BL WHO-FC (go with higher, switch to #)	BL 6MWD (meters)	O2 Req with walk test (L/min)	BL NT-proBNP/BNP	BL RV ECHO	TAPSE	RV Size	RV normal (1 = yes, 0 = no)	RV mild	RV mod	RV severe	RV Function	RV fxn normal	RV fxn mild	RV fxn mod	RV fxn severe	RVSP normal <35 (1 = normal, 0 = not normal)	RVSP (not reported) (1 = not reported)	RVSP high with #	EF (%) - take average if 2 numbers	BL RHC	RA (mean only)	RV systolic	RV diastolic	RV mean	PA systolic	PA diastolic	PA mean	PCWP (mean only)	CO (CI) thermodilution	CI (thermodilution)	PVR (woods)	Current PH meds	On baseline PH meds (0 = no, 1 = yes)	Bosetan (0 = no, 1 = yes)	Opsynvi (0 = no, 1 = yes)	Tyvaso (0 = no, 1 = yes)	Sildenafil (0 = no, 1 = yes)	Tadalafil (0 = no, 1 = yes)	Macitentan (0 = no, 1 = yes)	Selexipag (0 = no, 1 = yes)	Riociguat (0 = no, 1 = yes)	Sotatercept (0 = no, 1 = yes)	O2 Req (L/min)	Prior inhaler tyvaso DPI (0 = no, 1 = yes)	Prior inhaled Tyvaso nebs (0 = no, 1 = yes)	Reason for starting: new patient started on Yutrepia (0 = no, 1= yes)	Reason for starting: before Yutrepia shortness of breath (0 = no, 1= yes)	Reason for starting: cough 2/2 Tyvaso (0 = no, 1 = yes)	Reason for starting: throat irritation 2/2 Tyvaso (0 = no, 1 = yes)	Reason for starting: lack of dose optimization (0 = no, 1 = yes)	Yutrepia start date (YYYY-MM-DD)	Initial Yutrepia dose (mcg/session, inh/day, # carts/caps)	Titration schedule (dates and doses at each titration step)	Comment on row above	Maximum/maintenance Yutrepia dose (mcg/session and times/day, date achieved), assume QID (if BID, halve the number)	Maintenance date achieved	Days to maintenance date achieved	Device issues (mechanical issue) (1 = yes, 0 = no issue)		AE (cough, throat irritation, headache, dizziness, chest pain; severity, date onset)	Any AE (0 = no, 1 = yes)	Headache/dizziness	Fatigue/malaise	Shortness of breath	Hypoxemia	Nausea	Hypotension	Hypertension	Tachycardia	Cough (0 = no, 1 = yes)	Throat irritation	Cough details (onset date, severity 0-10, antitussive use, dose reduction bc of cough)	Dose changes due to AE (1 = yes, 0 = no)	Dose changes due to airway-related AE (1 = yes, 0 = no)	Stopped due adverse effect (1 = yes, 0 = no)	Stopped due airway-related AE (1 = yes, 0 = no)	DC of Yutrepia due to inefficacy (0 = no, 1 = yes)	DC of Yutrepia for any reason (0 = no, 1 = yes)	DC date of Yutrepia	Current date if no DC	Yutrepia duration if DC	Total duration on Yutrepia to current date)	Hospitalizations after start of Yutrepia (# and dates, reason-PAH worsening, safety/clinical AE, infection)	ER visits after start	FU 1m data (WHO-FC, 6MWD, NT-proBNP, AEs, early response)				FU 3-month data		FU 3-month BNP	FU 3-month 6MWT (distance)	TAPSE	RV size	RV normal (1 = yes, 0 = no)	RV mild	RV moderate	RV severe	RV function	RV fxn normal	RV fxn mild	RV fxn moderate	RV fxn severe	RVSP normal <35 (1 = normal, 0 = not normal)	RVSP (not reported) (1 = not reported)	RVSP high with #	EF (%) - take average if 2 numbers	BL RHC	RA (mean only)	RV systolic	RV diastolic	RV mean	PA systolic	PA diastolic	PA mean	PCWP (mean only)	CO (CI) thermodilution	CI (thermodilution)	PVR (woods)	FU 6-month data	FU 12-month data	Any repeat RHC after start (date and values of change)							Escalation to parenteral PCA (Y/N, date)	Lung transplant/death (Y/N, date, cause hard outcomes)
